# Growth and dry matter partitioning response in cereal-legume intercropping under full and limited irrigation regimes

**DOI:** 10.1038/s41598-021-92022-4

**Published:** 2021-06-15

**Authors:** Amanullah, Shah Khalid, Farhan Khalil, Mohamed Soliman Elshikh, Mona S. Alwahibi, Jawaher Alkahtani, Imranuddin, Imran

**Affiliations:** 1 Department of Agronomy, The University of Agriculture Peshawar, Peshawar, Pakistan; 2 Department of Botany and Microbiology, College of Science, King Saud University, Riyadh, 11451 Saudi Arabia

**Keywords:** Plant sciences, Environmental sciences

## Abstract

The dry matter partitioning is the product of the flow of assimilates from the source organs (leaves and stems) along the transport route to the storage organs (grains). A 2-year field experiment was conducted at the agronomy research farm of the University of Agriculture Peshawar, Pakistan during 2015–2016 (Y1) to 2016–2017 (Y2) having semiarid climate. Four summer crops, pearl millet (*Pennisetum typhoidum* L.), sorghum (*Sorghum bicolor* L.) and mungbean (*Vigna radiata* L.) and pigeonpea (*Cajanus cajan* L.) and four winter crops, wheat (*Triticum aestivum* L.), barley (*Hordeum vulgare* L.), fababean (*Vicia faba*) and rapeseed (*Brassica napus*) were grown under two irrigation regimes (full vs. limited irrigation) with the pattern of growing each crop either alone as sole crop or in combination of two crops in each intercropping system under both winter and summer seasons. The result showed that under full irrigated condition (no water stress), all crops had higher crop growth rate (CGR), leaf dry weight (LDW), stem dry weight (SDW), and spike/head dry weight (S/H/PDW) at both anthesis and physiological maturity (PM) than limited irrigated condition (water stress). In winter crops, both wheat and barley grown as sole crop or intercropped with fababean produced maximum CGR, LDW, SDW, S/H/PDW than other intercrops. Among summer crops, sorghum intercropped either with pigeon pea or with mungbean produced maximum CGR, LDW, SDW, and S/H/PDW at both growth stages. Sole mungbean and pigeon pea or pigeon pea and mungbean intercropping had higher CGR, LDW, SDW, S/H/PDW than millet and sorghum intercropping. On the other hand, wheat and barley grown as sole crops or intercropped with fababean produced maximum CGR, LDW, SDW, and S/H/PDW than other intercrops. Fababean grown as sole crop or intercropped with wheat produced higher CGR, LDW, SDW, and S/H/PDW at PM than intercropped with barley or rapeseed. From the results it was concluded that cereal plus legume intercropping particularly wheat/fababean in winter and sorghum/pigeon pea or sorgum/mungbean in summer are the most productive intercropping systems under both low and high moisture regimes.

## Introduction

One approach to optimize yield is to change distribution of dry matter (DM) of plants between roots and shoots^1^. Changing the root distribution of DM can increase the reproductive secretions of plants, which may be beneficial for increasing yields^2^. To improve the adaptive capacity of crops to drought (water stress), the distribution of DM can be coordinated between roots and shoots^3^. Straw retention improves soil organic carbon content and the amount depends on soil types, climatic conditions and management strategies^4–6^. Annual carbon input to soil from crop residues can be divided into two main sources: above ground (i.e. straw, stubble and surface debris) and below ground (i.e. root biomass remaining in soil at harvest, root turnover, exudates and excretions). One of several suggested management methods capture atmospheric carbon dioxide (CO_2_), as the organic weight of the soil must increase the area under crops^6^. The ratio of shoots to roots by weight gives an estimate of the mass of roots that remains in the soil if the shoots weight is known and the DM distribution in the roots is large at the germination stage and steadily decreases throughout development^7^. Different varieties of wheat can have the same depth of growth or rooting above ground, but differ in root biomass^8^. The shoot-to-root ratio in different crops increases with age^9^, and environmental stresses increase the relative mass of roots compared to shoots^10^. In addition, intercropping legumes with cereals provides higher land-use efficiency, less water consumption, and more environmental benefits over cereal mono-crops (sole crops). Intercropping often involve interspecies assistance and inter-specific competition^11–13^. However, there is a great advantage in yields when sowing together in comparison with the corresponding single (sole) crops^14–16^. This is largely because one component can improve the survival, growth, or fitness of another component^17^. Therefore, one component can affect the operation of other components in the whole farming system.

Cereal-legumes intercropping system can increase yield of crops by sharing the same available environmental resources, intercropping also increase crop productivity as compared to sole cropping^18–20^. Marer, et al.^21^ reported intercropping advantages over mono-cropping. For the intercropping of leguminous crops and non-N-fixing crops (e.g. cereal crops), Willey^22^ proposed that the potential advantages of intercropping with legumes are: increasing the total yield by increasing the land equivalent ratio, increasing the effective N content in the soil and reducing the effect of N transfer on chemical N fertilizers. Increase in the use efficiency of water, N and other macro and micronutrients; reduce costs of crop production and market risks through crop diversification and reduce pests damage. Drought^23^ is a significant limiting factor for agricultural productivity and tends to inhibit plant growth by reducing water absorption and nutrient absorption. Reduced water availability usually results in decreased growth and final crop yields. However, plant species in a mixed growing system can differ in their response to growth under water scarcity conditions, since water availability is known to be spatially heterogeneous and distributed over time and space^24,25^. The current challenge in agriculture is to increase yields by using less water, especially in regions with limited land and water resources^26^. Efficient irrigation systems require the selection of an appropriate method for crop growth, adequate monitoring of the irrigation system and water supply, and appropriate application rates depending on the stage of crop growth. Watering requirements vary by location, soil type, and cultural practice^27^. There is lack of research on DM partitioning into plants parts (roots, stems, leaves, shoots) in winter and summer cereal and legumes intercropping system under different water regimes. The objective of this study was to investigate the differences in DM partitioning into different plant parts among the winter and summer season crops grown under full (03 irrigations having no water stress) and limited (01 irrigation having water stress) irrigations regimes.

## Materials and methods

### Field experiment

“A 2-year field experiments were conducted during 2015–2016 (Y1) and 2016–2017 (Y2) at the Agronomy Research Farm of the University of Agriculture Peshawar, Pakistan. The experimental site has a continental climate and is located at 34° 27′ 12.46″ N latitude and 71° 27′56.4″ E longitude with altitude of 359 m above sea level having semiarid climate. Two adjacent fields were used separated by one meter in each year viz. one under limited irrigation (water stress) and the second one under full irrigation (no water stress), both fields had similar physiochemical properties. The experiment under each irrigation regime was conducted in a randomized complete block design (combined over-irrigation) having four replications. A subplot size of 4 m × 4 m was used. Each plot was separated by a 0.5 m earthen band to prevent the flow of water and mobile nutrients to nearby plots. A recommended basal dose of nitrogen (N) and phosphorus (P) for cereals was 120 kg N and 60 kg P ha^−1^, while in the case of legumes crops, 30 kg N and 60 kg P ha^−1^ were used, respectively. DAP (Di-ammonium phosphate) was used as a source of P and N, while the remaining N was applied through urea. In the case of fababean, rapeseed, mungbean, and pigeon pea all N (30 kg ha^−1^) was applied at sowing time, while for cereal crops N was applied in two equal splits (60 kg ha^−1^ at sowing time and 60 kg ha^−1^ at the tillering stage). Phosphorus at the rate of 60 kg P ha^−1^ in the form of DAP was applied. Adjustment of N and P from DAP and urea were made. The required P was applied at the time of seedbed preparation. All other agronomic practices were kept normal and uniform for all the treatments^28^. In each intercropping system two crops were sown in alternate manner. Pre-experimentation soil physiochemical properties of the experimental site was silty clay loam in texture with concentration of clay (31.23%), silt (51.5%), extractable phosphorus (6.57 mg kg^−1^), extractable zinc (0.7 mg kg^−1^), total nitrogen (0.04%), organic carbon (0.87%), and soil pH (7.8).

#### Factor A. irrigation

1. **Limited irrigation**: only one irrigation (75 mm) was applied at booting stage of wheat to the winter crops, while in the case of summer crops irrigations were given at pre-sowing and at the anthesis stage of pearl millet.
2. **Full irrigation**: three irrigations, at tillering (95 mm), jointing (92 mm) and booting stage (75 mm) of wheat were applied to the winter crops, while in case of summer crops irrigation was applied at pre-sowing, stem elongation, anthesis, and dough stage of pearl millet^28^.

To calculate the amount of water applied at each irrigation (Float cut method) of Misra and Ahmad^29^ was applied.

### Experiment one: Four winter crops (wheat, barley, rapeseed & fababean)

#### Factor B. Intercropping system (winter crops)

1. Wheat sole crop
2. Barley sole crop
3. Fababean sole crop
4. Rapeseed sole crop
5. Wheat + barley
6. Wheat + fababean
7. Wheat + rapeseed
8. Barley + fababean
9. Barley + rapeseed
10. Fababean + rapeseed

### Experiment two: Four summer crops (sorghum, pearl millet, mungbean & pigeonpea)

#### Intercropping system (summer crops)

1. Sorghum sole crop
2. Pearl millet sole crop
3. Mungbean sole crop
4. Pigeonpea sole crop
5. Sorghum + pearl millet
6. Sorghum + mungbean
7. Sorghum + pigeonpea
8. Pearl millet + mungbean
9. Pearl millet + pigeonpea
10. Mungbean + pigeonpea

Data were recorded according to the formulas proposed by Moll et al.^30^ and Ortiz‐Monasterio et al.^31^. For determination of dry matter (DM) partitioning into various plant parts, a random sample was taken of the above-ground part of the plant from each plot at physiological maturity and separated into the stems, leaves, and heads. The materials was put in paper bags and allowed to dry at 60 °C in the oven for 72 h to become dry and achieve constant weight. The samples were weighed using the electronic balance and the average data on DM of leaves, stems, spikes, heads, and pods was worked out. Crop growth rate (CGR), which is DM accumulation per unit area per unit time was determined using the following formula:

$${\text{CGR }} = {\text{ W}}_{{2}} - {\text{ W}}_{{1}} / \, \left( {{\text{t}}_{{2}} - {\text{ t}}_{{1}} } \right) \, \left( {{\text{g m}}^{{ - {2}}} {\text{day}}^{{ - {1}}} } \right)$$

where *W*_1_ = dry weight per plant at the beginning of interval; *W*_2_ = dry weight per plant at the end of interval; *t*_2_ *−* *t*_1_ = the time interval between the two consecutive sampling.

## Results

### Winter crops

#### Crop growth rate (g m^−2^ day^−1^)

**Figure 1:**
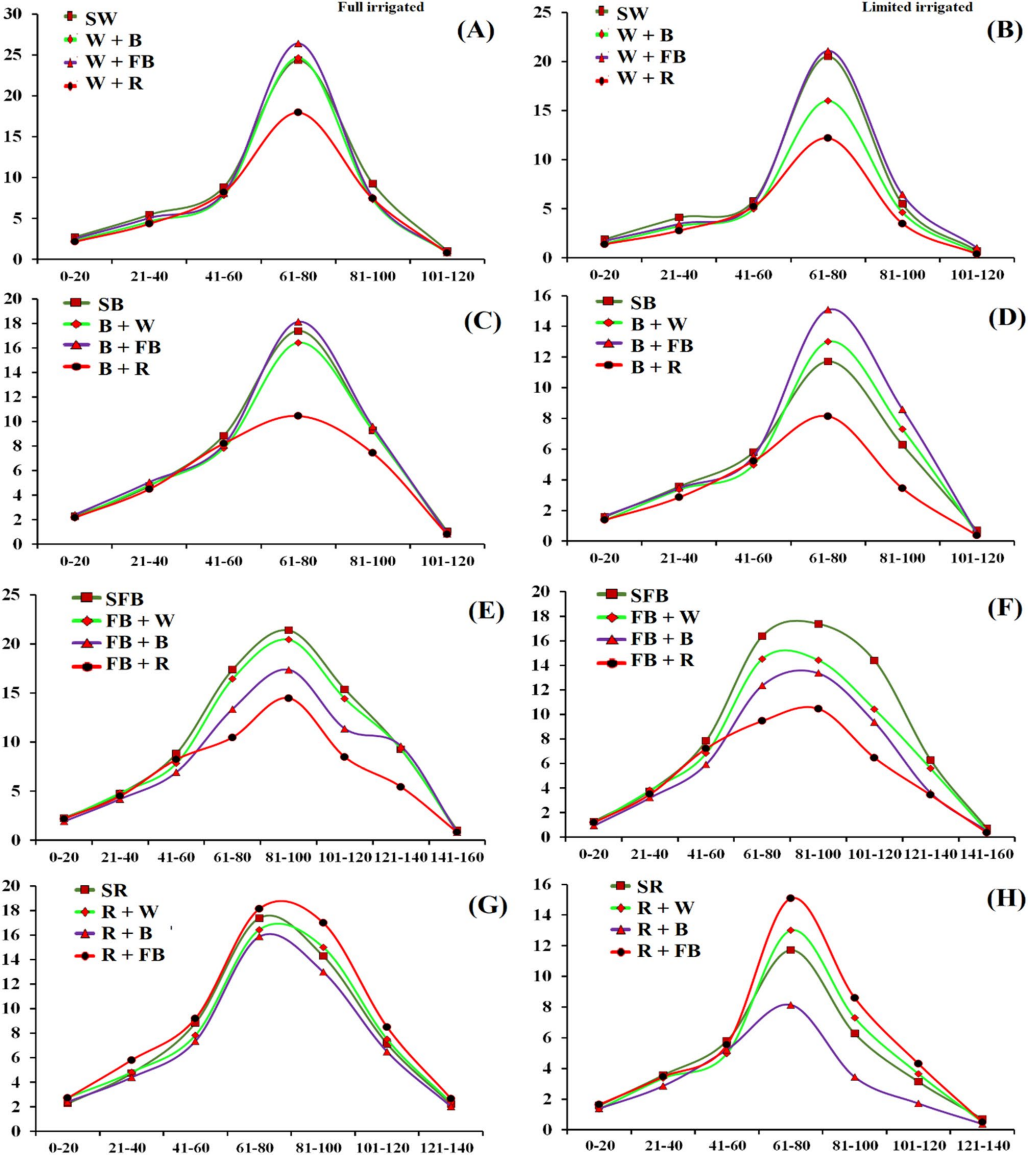
Crop growth rate of winter cereal and legumes crops as affected by intercropping and irrigation regimes. W, B, FB and R, stand for wheat, barley, fababean and rapeseed, respectively.

Crop growth rate of wheat under various intercropping systems was significantly different under different water regimes. Maximum CGR was recorded for sole wheat and wheat intercropped with fababean (Fig. 1A) while minimum was recorded when wheat was intercropped with rapeseed under both full and limited water regimes. Figure 1B shows that rapeseed and barley have a strong competitive ability against wheat under limited water supply. Both barley and rapeseed have strong and deep root system than wheat, as result acquired more water and nutrients than wheat under scare resources. Barley intercropping system showed that under both water regimes barley intercropped with fababean increased CGR of barley. Maximum CGR was recorded for barley intercropped with fababean and wheat while lowest was recorded for barley intercropped with rapeseed under both water regimes (Fig. 1C,D). However, rapeseed have a strongly influence on the CGR of wheat under both water regimes. In the case of fababean, maximum CGR was recorded for sole fababean and fababean intercropped with wheat under both water regimes (Fig. 1E,F). Intercropping of fababean and wheat proved to be the most compatible cropping system as compared with fababean intercropped with barley or rapeseed. In case of rapeseed, maximum CGR was recorded for rapeseed when intercropped with fababean under both water regimes (Fig. 1G,H), however under full irrigated condition CGR was higher than limited irrigated condition. Under limited irrigated condition, intercropping of barley with rapeseed decreased CGR of rapeseed, which showed a strong competitive ability of barley for nutrient and water acquisition.

**Figure 2:**
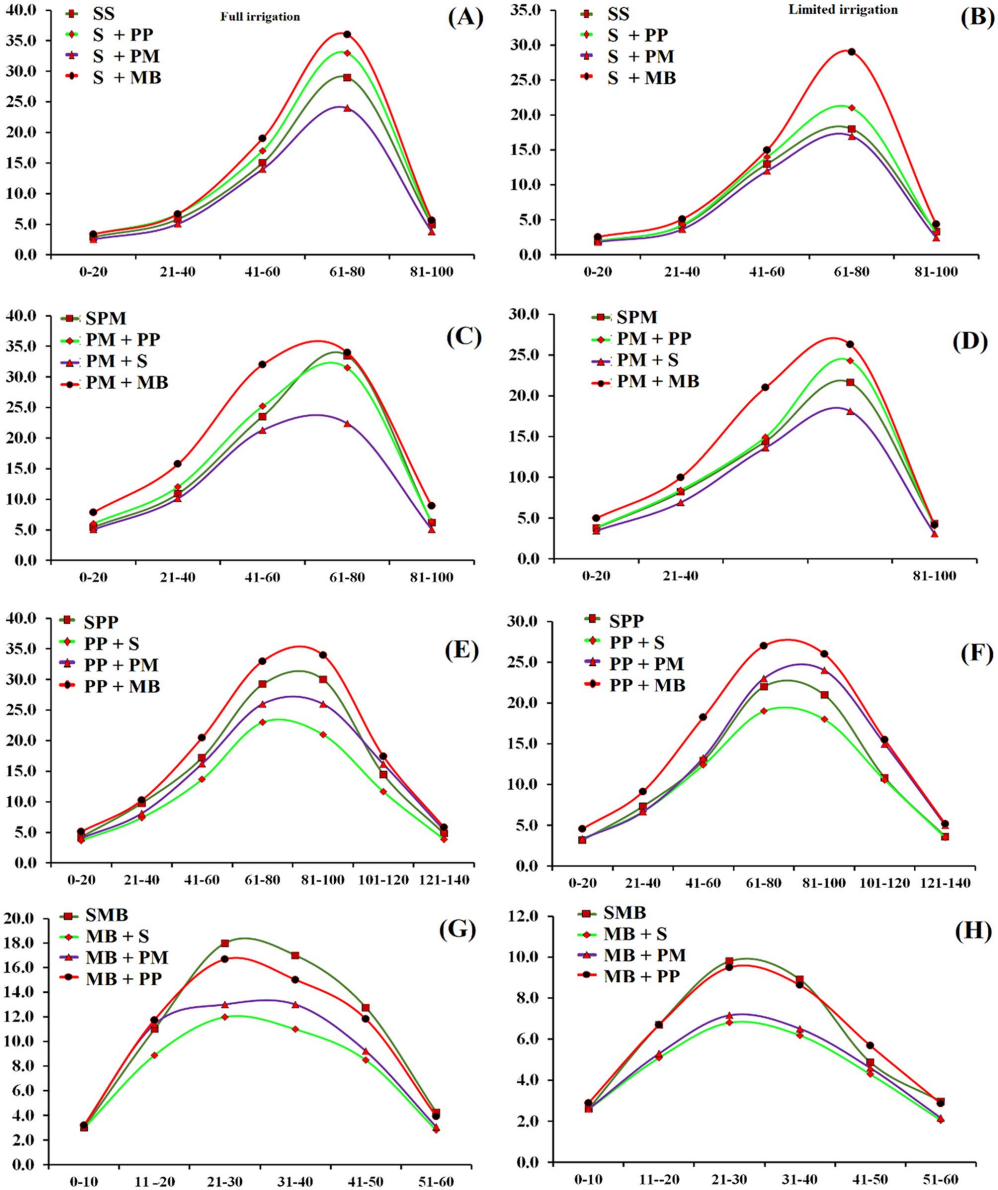
Crop growth rate of summer cereal and legumes crops as affected by intercropping and irrigation regimes. PM, MB, S and PP stand for millet, mungbean, sorghum and pigeon pea, respectively.

The CGR of summer crops i.e., sorghum, pearl millet, mungbean and pigeonpea as affected by intercropping and irrigation regime are shown in Fig. 2A,B,C,D, respectively. All the crops showed higher CGR under full irrigated condition than limited irrigation. Sorghum intercropped with mungbean produced higher CGR under both water regimes, while sorghum intercropped with pearl millet or grown as sole crop showed the least CGR. The figures revealed that sorghum intercropped with both legumes crops, increased the CGR of sorghum than sole sorghum or sorghum intercropped with pearl millet (Fig. 2A,B). Similarly, pearl millet intercropped either with mungbean and pigeonpea produced higher CGR than grown as sole crop or in combination with sorghum (Fig. 2C,D). Moreover, pigeonpea produced the highest CGR while grown as sole crop or intercropped with mungbean, while lowest CGR was recorded when pigeonpea was intercropped either with sorghum and pearl millet (Fig. 2E,F). In case of mungbean, higher CGR was recorded for sole mungbean and when intercropped with pigeonpea while the lowest CGR was recorded when mungbean was intercropped with sorghum and pearl millet under both water regimes (Fig. 2G,H).

#### Leaf dry weight of winter crops (g m^−2^) at anthesis stage

**Figure3:**
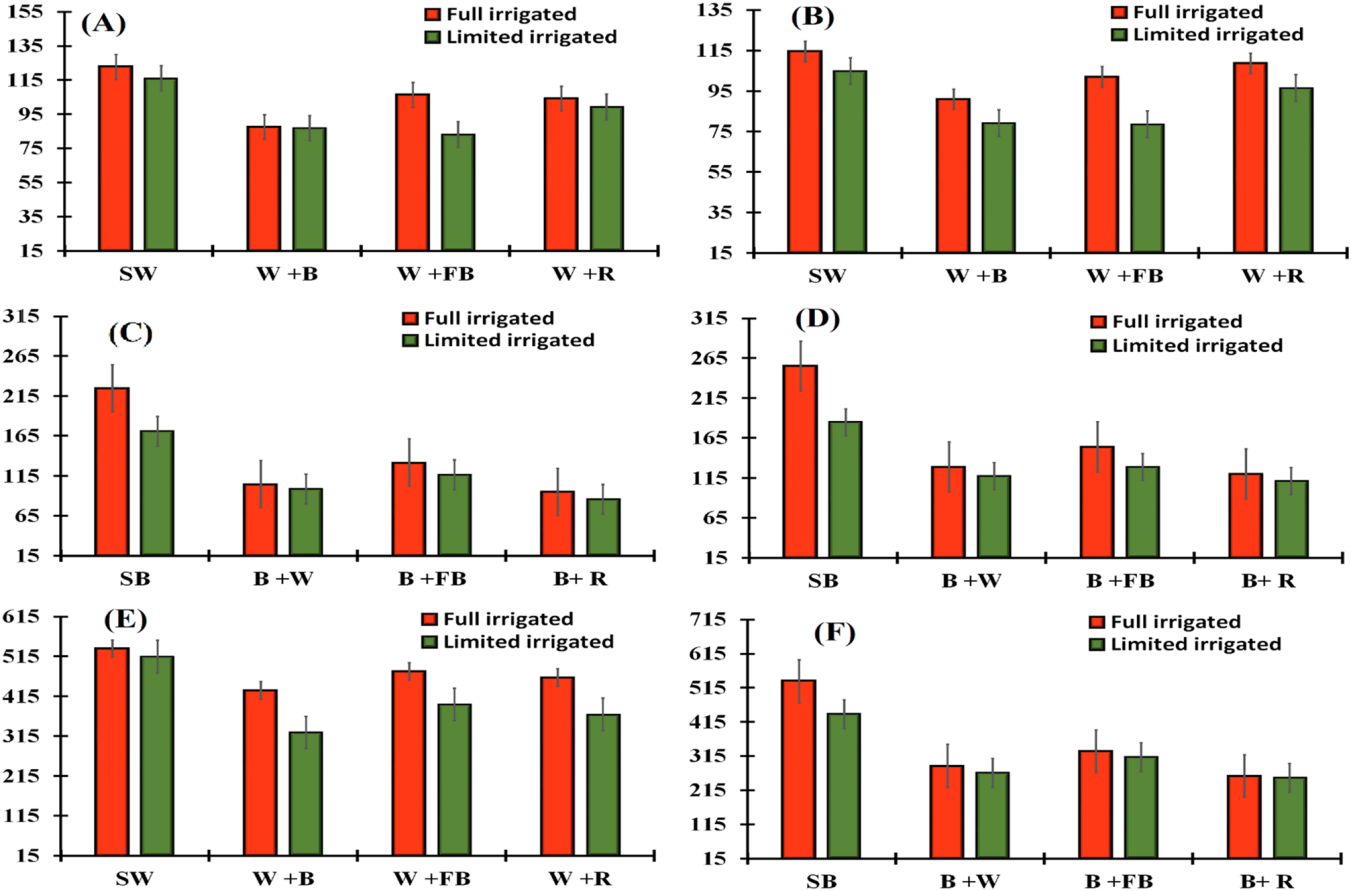
Leaves and stem dry weight of barley (**A**) and (**B**), respectively, at anthesis, leaves and stem dry weight of barely (**C**,**D**), respectively, and spike dry weight of wheat and barley (**E**,**F**), respectively at physiological maturity.

All crops grown under full irrigated condition produced higher LDW at anthesis than limited irrigated condition (Table 1). Both cereals (wheat or barley) intercropped with fababean produced maximum LDW at anthesis than intercropped with rapeseed (Table 2A,B), respectively. Among intercropping, pure stands (sole crops) had produced higher LDW at anthesis when compared to other intercropping, followed by fababean + wheat, and fababean + rapeseed. Among interactions, wheat + fababean had the highest production of LDW with full irrigation supply, while the lowest LDW was obtained under limited irrigation for wheat + rapeseed intercrop (Fig. 3A).The planned mean comparison specified that wheat/barley intercropped with fababean produced maximum LDW m^−2^ at anthesis than barley and wheat intercropped with each other. On the other hand, fababean intercropped with barley/wheat produced less LDW m^−2^ at anthesis than fababean intercropped with rapeseed (Table 2C). Moreover, wheat and/or barley intercropped with fababean produced the higher LDW m^−2^ at anthesis than intercropped with rapeseed (Table 2D).

**Table 1:** Dry matter partitioning (g m^−2^) at anthesis of winter crops as affected by intercropping and irrigation regimes.

Intercropping	Leaf	Stem	Head	Intercropping	Leaf	Stem	Head
Sole Wheat	199.3 a	109.6 a	278.4 a	Sole Barley	114.7 a	101.8 a	258.4 a
Wheat + Barley	87.3 d	84.9 d	195.5 c	Wheat + Barley	60.9 c	58.0 c	127.4 c
Wheat + Fababean	94.6 c	9.34 c	214.5 b	Barley + Fababean	75.6 b	71.4 b	150.5 b
Wheat + Brassica	101.7 b	102.5 b	225.5 b	Barley + Brassica	57.1 d	57.3 c	125.9 c
Full irrigation	105.2 a	104.7 a	243.2 a	full irrigation	81.7 a	77.9 a	179.5 a
Limited irrigation	96.3 b	89.7 b	213.5 b	Limited irrigation	72.4 b	66.3 b	151.6 b
LSD 0.05 Irrigation	0.0	0.0	0.0	LSD 0.05 Irrigation	0.0	0.0	0.0
LSD 0.05 I.C	5.5	4.4	11.0	LSD 0.05 I.C	2.7	3.0	5.3
LSD 0.05 I × I.C	7.5	7.4	16.0	LSD 0.05 I × I.C	3.7	5.0	8.3
Sole Fababean	121.7 a	163.1 a	114.6 a	Sole Rapeseed	130.1 a	149.1 a	129.1 a
Fababean + Wheat	103.8 b	89.1 c	107.5 b	Rapeseed + Wheat	68.4 b	80.1 c	60.1 c
Fababean + Barley	87.9 c	84.5 c	88.9 d	Rapeseed + Barley	51.5 d	75.5 d	55.5 d
Fababean + Rapeseed	101.6 b	101.4 b	98.0 c	Rapeseed + Fababean	56.1 c	92.4 b	72.4 b
full irrigation	107.6 a	118.1 a	108.9 a	full irrigation	85.1 a	109.1 a	89.1 a
Limited irrigation	99.9 b	101.0 b	95.6 b	Limited irrigation	72.5 b	89.5 b	69.5 b
LSD 0.05 Irrigation	0.0	0.0	0.0	LSD 0.05 Irrigation	0.0	0.0	0.0
LSD 0.05 I.C	4.5	5.7	5.4	LSD for intercropping	1.4	11.7	11.7
LSD 0.05 I × I.C	7.5	8.0	7.5	LSD for interaction	1.9	16.6	16.6

**Table 2:** Pre-planned comparison of different intercropping systems at anthesis of winter crops as affected by intercropping and irrigation regimes.

**Leaves Dry weight (g) m**^**−2**^	Wheat **(A)**	Sole Vs	Intercrop	Sig:	W + FB Vs	W + B	Sig:	W + FB Vs	W + B/R	Sig:
		119.33	94.55	*	94.64	87.29	**	101.71	90.97	*
	Barley **(B)**	Sole Vs	Intercrop	Sig:	B + FB Vs	W + B	Sig:	B + FB Vs	B + W/R	Sig:
		114.69	64.53	*	75.6	60.92	*	75.6	68.26	*
	Fababean **(C)**	Sole Vs	Intercrop	Sig:	FB + B Vs	FB + W	Sig:	FB + R	FB + W/B	Sig:
		122	98	**	88	102	**	104	95	**
	Rapeseed **(D)**	Sole Vs	Intercrop	Sig:	R + B Vs	R + W	Sig:	R + FB Vs	R + W/B	Sig:
		130	59	**	52	56	**	68	54	**
**Stem Dry weight (g) m**^**−2**^	Wheat **(E)**	Sole Vs	Intercrop	Sig:	W + FB Vs	W + B	Sig:	W + FBVs	W + B/R	Sig:
		109.66	92.63	*	90.39	84.98	*	90.39	87.68	*
	Barley **(F)**	Sole Vs	Intercrop	Sig:	B + FB Vs	B + W	Sig:	B + FB Vs	B + W/R	Sig:
		101.8	62.23	*	71.37	58.01	*	71.37	64.69	*
	Fababean **(G)**	Sole Vs	Intercrop	Sig:	FB + B Vs	FB + W	Sig:	FB + R	FB + W/B	Sig:
		163	92	**	85	101	**	89	93	Ns
	Rapeseed **(H)**	Sole Vs	Intercrop	Sig:	R + B Vs	R + W	Sig:	R + FB Vs	R + W/B	Sig:
		149	83	**	76	92	**	80	84	Ns
**Spike/Pod Dry weight (g) m**^**−2**^	Wheat **(I)**	Sole Vs	Intercrop	Sig:	W + FB Vs	W + B	Sig:	W + R Vs	( W + FB/R)	Sig:
		278.04	211.83	*	214.51	195.51	**	225.46	205.01	*
	Barley **(J)**	Sole Vs	Intercrop	Sig:	B + FB Vs	W + B	Sig:	B + R Vs	B + FB/R	Sig:
		258.42	134.65	*	150.55	127.42	*	125.99	138.98	*
	Fababean **(K)**	Sole Vs	Intercrop	Sig:	FB + B Vs	FB + W	Sig:	FB + R vs	FB + W/B	Sig:
		115	98	**	89	98	**	108	93	Ns
	Rapeseed **(L)**	Sole Vs	Intercrop	Sig:	R + B Vs	R + W	Sig:	R + FB Vs	R + W/B	Sig:
		129	63	**	56	72	**	60	64	Ns

Where *, ** stands for significant at 5 and 1% level of probability, respectively. W, B, FB and R, stand for wheat, barley, fababean and rapeseed, respectively. Means in the same category are not significantly different if followed by at least one common letter at (*P* ≤ 0.05) level.

#### Stem dry weight of winter crops (g m^−2^) at anthesis stage

Data regarding stem dry weight (SDW) (g m^−2^) is presented in Table 1. Higher SDW at anthesis was recorded under full irrigation than limited irrigation. Cereal crops (barley or wheat) intercropped with fababean produced maximum SDW (g m^−2^) at anthesis, than when intercropped with each other (Table 2E,F), respectively. Wheat and barley intercropped with fababean produced higher SDW m^−2^ at anthesis, followed by intercropped with rapeseed, while the lowest SDW plant^−1^ for both wheat and barley were observed when intercropped with each other. Fababean sown as sole crop produced higher SDW plant^−1^ at anthesis than intercropped with wheat/barley (Table 2G), respectively. Interaction of irrigation and intercropping had statistically significant effects on SDW at anthesis stage and maximum SDW was reported for monoculture of cereals with full irrigation. Likewise, minimum SDW was shown by wheat + fababean with limited irrigation supply (Fig. 3B). The planned mean comparison quantified that fababean intercropped with wheat/barley produced maximum SDW m^−2^ at anthesis than barley and wheat intercropped with each other. Moreover, fababean intercropped with barley/wheat produced higher SDW m^−2^ at anthesis than fababean intercropped with rapeseed (Table 2H).

#### Spike/pod dry weight of winter crops (g m^−2^) at anthesis stage

Data regarding spike/pod dry weight (S/PDW) of wheat, barley, fababean and rapeseed are presented in Table 1. Higher S/PDW for all crops at anthesis was recorded under full irrigation than limited irrigation. Moreover, in the case of intercropping, sole fababean produced more pod dry weight compared to other intercrops, followed by fababean + wheat and wheat + rapeseed, while the lowest S/PDW was recorded for fababean + barley. The planned mean comparison quantified that fababean intercropped with wheat/barley produced maximum S/PDW m^−2^ at anthesis than barley and wheat intercropped with each other (Table 2I,J). Moreover, fababean intercropped with barley/wheat produced less S/PDW m^−2^ at anthesis than fababean intercropped with rapeseed (Table 2K). Rapeseed intercropped with wheat and/or barley produced higher SDW m^−2^ at anthesis than intercropped with rapeseed (Table 2L).

#### Leaf dry weight (g m^−2^) at physiological maturity

Data about leaf dry weight (LDW) of wheat, barley, fababean and rapeseed at PM are shown in Table 3. Both irrigation and intercropping significantly affected LDW. All crops grown under full irrigated condition produced higher LDW than limited irrigated condition. Wheat and barley intercropped with fababean or rapeseed produced maximum LDW than when both crops were intercropped with each other (Table 4A,B), respectively. Fababean grown as sole crops or intercropped with wheat produced higher LDW than intercropped with barley (Table 4C). Rapeseed grown as a sole crop or intercropped with fababean produced higher LDW than intercropped with barley/wheat (Table 4D). Interaction of irrigation and intercropping had statistically significant effects on LDW of barley where maximum LDW was reported for monoculture barely under full irrigation. Likewise, minimum weight given by barely + rapeseed with limited irrigation supply (Fig. 3C). The planned mean comparison specified that wheat/barley intercropped with fababean produced higher LDW than intercropped with rapeseed (Table 4A,B).

**Table 3:** Dry matter partitioning (g m^−2^) at physiological maturity of winter crops as affected by intercropping and irrigation regimes.

Intercropping	Leaf	Stem	Head	Intercropping	Leaf	Stem	Head
Sole Wheat	202.4 a	270.0 a	524.3 a	Sole Barley	197.5 a	219.7 a	485.7 a
Wheat + Barley	144.9 c	160.6 d	376.9 d	Wheat + Barley	101.6 c	123.2 c	276.1 c
Wheat + Fababean	158.1 b	202.9 b	435.9 b	Barley + Fababean	123.9 b	141.4 b	320.3 b
Wheat + Brassica	165.5 b	192.4 c	415.5 c	Barley + Brassica	90.5 d	115.6 d	253.8 d
Full irrigation	174.7 a	215.8 a	475.6 a	full irrigation	138.9 a	164.5 a	351.6 a
Limited irrigation	160.7 b	197.1 b	400.6 b	Limited irrigation	117.8 b	135.5 b	316.3 b
LSD 0.05 Irrigation	*	*	*	LSD 0.05 Irrigation	*	*	*
LSD 0.05 I.C	8.1	3.8	12.3	LSD 0.05 I.C	3.8	9.3	18.2
LSD 0.05 I × I.C	15.3	6.8	22.3	LSD 0.05 I × I.C	5.8	16.3	30.2
Sole Fababean	103.6 a	303.1 a	332.4 a	Sole Rapeseed	163 a	299.5 a	345.6 a
Fababean + Wheat	96.5 b	229.1 b	311.7 b	Rapeseed + Wheat	94 c	234.5 b	320.2 b
Fababean + Barley	77.2 d	241.3b	257.7 d	Rapeseed + Barley	90 d	213.5 c	312.8 b
Fababean + Rapeseed	87.5 c	224.5c	284.2 c	Rapeseed + Fababean	106 b	242.3 b	339.8 a
full irrigation	97.9 a	258.0 a	315.9 a	full irrigation	133 a	259.0 a	341.5 a
Limited irrigation	84.5 b	241 b	277.1 b	Limited irrigation	111 b	239.5 b	317.7 b
irrigation	*	*	*		*	*	*
LSD for Intercropping	3.8	4.7	10.4	LSD for intercropping	4.4	11.7	14.2
LSD for Interaction	5.5	7.4	14.5	LSD for interaction	7.9	16.3	19.5

**Table 4:** Pre-planned comparison of different intercropping systems at physiological maturity of winter crops as affected by intercropping and irrigation regimes.

**Leaves Dry weight (g) m**^**−2**^	Wheat **(A)**	Sole Vs	Intercrop	Sig:	W + FB Vs	W + B	Sig:	W + R Vs	( W + FB/R)	Sig:
		202.35	156.19	*	158.12	144.99	**	165.46	151.55	*
	Barley **(B)**	Sole Vs	Intercrop	Sig:	B + FB Vs	W + B	Sig:	B + R Vs	B + FB/W	Sig:
		197.49	105.36	*	123.97	101.66	*	90.45	112.82	*
	Fababean **(C)**	Sole Vs	Intercrop	Sig:	FB + B Vs	FB + W	Sig:	FB + R	FB + W/B	Sig:
		104	87	**	78	87	**	97	82	**
	Rapeseed **(D)**	Sole Vs	Intercrop	Sig:	R + B Vs	R + W	Sig:	R + FB Vs	R + W/B	Sig:
		163	97	**	90	106	**	94	98	**
**Stem Dry weight (g) m**^**−2**^	Wheat **(E)**	Sole Vs	Intercrop	Sig:	W + F Vs	W + B	Sig:	W + Br Vs	W + FB/R)	Sig:
		270	185.3	*	202.91	160.62	*	192.38	181.77	*
	Barley **(F)**	Sole Vs	Intercrop	Sig:	B + FB Vs	W + B	Sig:	B + Br Vs	B + FB/R	Sig:
		219.73	126.76	*	141.4	123.23	*	115.64	132.32	*
	Fababean **(G)**	Sole Vs	Intercrop	Sig:	FB + B Vs	FB + W	Sig:	FB + R	FB + W/B	Sig:
		303	232	**	225	241	**	229	233	**
	Rapeseed **(H)**	Sole Vs	Intercrop	Sig:	R + B Vs	R + W	Sig:	R + FB Vs	R + W/B	Sig:
		300	230	**	214	242	**	235	228	**
**Spike/Pod Dry weight (g) m**^**−2**^	Wheat **(I)**	Sole Vs	Intercrop	Sig:	W + F Vs	W + B	Sig:	W + Br Vs	( W + FB/R)	Sig:
		524.27	409.41	*	435.88	376.86	*	415.5	406.37	*
	Barley **(J)**	Sole Vs	Intercrop	Sig:	B + FB Vs	W + B	Sig:	B + Br Vs	B + FB/R	Sig:
		485.69	283.37	**	320.28	276.05	*	253.79	298.17	*
	Fababean **(K)**	Sole Vs	Intercrop	Sig:	FB + B Vs	FB + W	Sig:	FB + R	FB + W/B	Sig:
		332	285	**	258	284	**	312	271	ns
	Rapeseed **(L)**	Sole Vs	Intercrop	Sig:	R + B Vs	R + W	Sig:	R + FB Vs	R + W/B	Sig:
		346	324	**	313	340	**	320	326	**

Where *, ** stands for significant at 5 and 1% level of probability, respectively. W, B, FB and R, stand for wheat, barley, fababean and rapeseed, respectively. Means in the same category are not significantly different if followed by at least one common letter at (*P* ≤ 0.05) level.

#### Stem dry weight (g m^−2^) at physiological maturity

Data regarding stem dry weight (SDW) of wheat, barley, fababean and rapeseed at PM are presented in Table 3. Under full irrigated condition, all crops had produced significantly higher SDW than limited irrigated condition. Both wheat and barley produced higher SDW while grown as sole crops, followed by intercropped with fababean, while the lowest SDW of both cereals were observed when intercropped with each other or intercropped with rapeseed (Table 4E,F), respectively. Fababean grown as sole or intercropped with wheat produced higher SDW than intercropped with barely and rapeseed (Table 4G). The planned mean comparison specified that cereals (wheat and barley) intercropped with fababean produced maximum SDW than intercropped with other cereals. In divergence, fababean intercropped with cereals produced high SDW than fababean intercropped with rapeseed. Moreover, rapeseed intercropped with fababean produced higher SDW than intercropped with wheat and barley (Table 4H). Interaction of irrigation and intercropping had statistically significant effects on SDW of barley and maximum SDW was reported for monoculture barely with full irrigation. Minimum SDW by barely + rapeseed was produced with limited irrigation supply (Fig. 3D).

#### Spike/pods dry weight (g m^−2^) at physiological maturity

Wheat, barley, fababean and rapeseed spike/pods dry weight (S/PDW) at PM are presented in Table 3. The S/PDW of all crops was significantly affected by irrigation and intercropping. All crops grown under full irrigated condition produced higher S/PDW than limited irrigated condition. Interaction of irrigation and intercropping had statistically significant effects on S/PDW of wheat and barley where maximum weight was reported for monoculture wheat and barley with full irrigation, likewise, minimum weight given by wheat and barley when intercropped with rapeseed under limited irrigation supply (Fig. 3E,F). Cereals, wheat and barley grown as sole crops or intercropped with fababean produced maximum S/PDW than other intercrops (Table 4I,J). Fababean grown as sole crop or intercropped with wheat produced higher S/PDW than intercropped with barley or rapeseed (Table 4K). In contrast, rapeseed intercropped with wheat produced higher S/PDW than rapeseed intercropped with barley (Table 4L).

### Summer crops

#### Leaf dry weight (g plant^−1^) at anthesis

**Figure 4:**
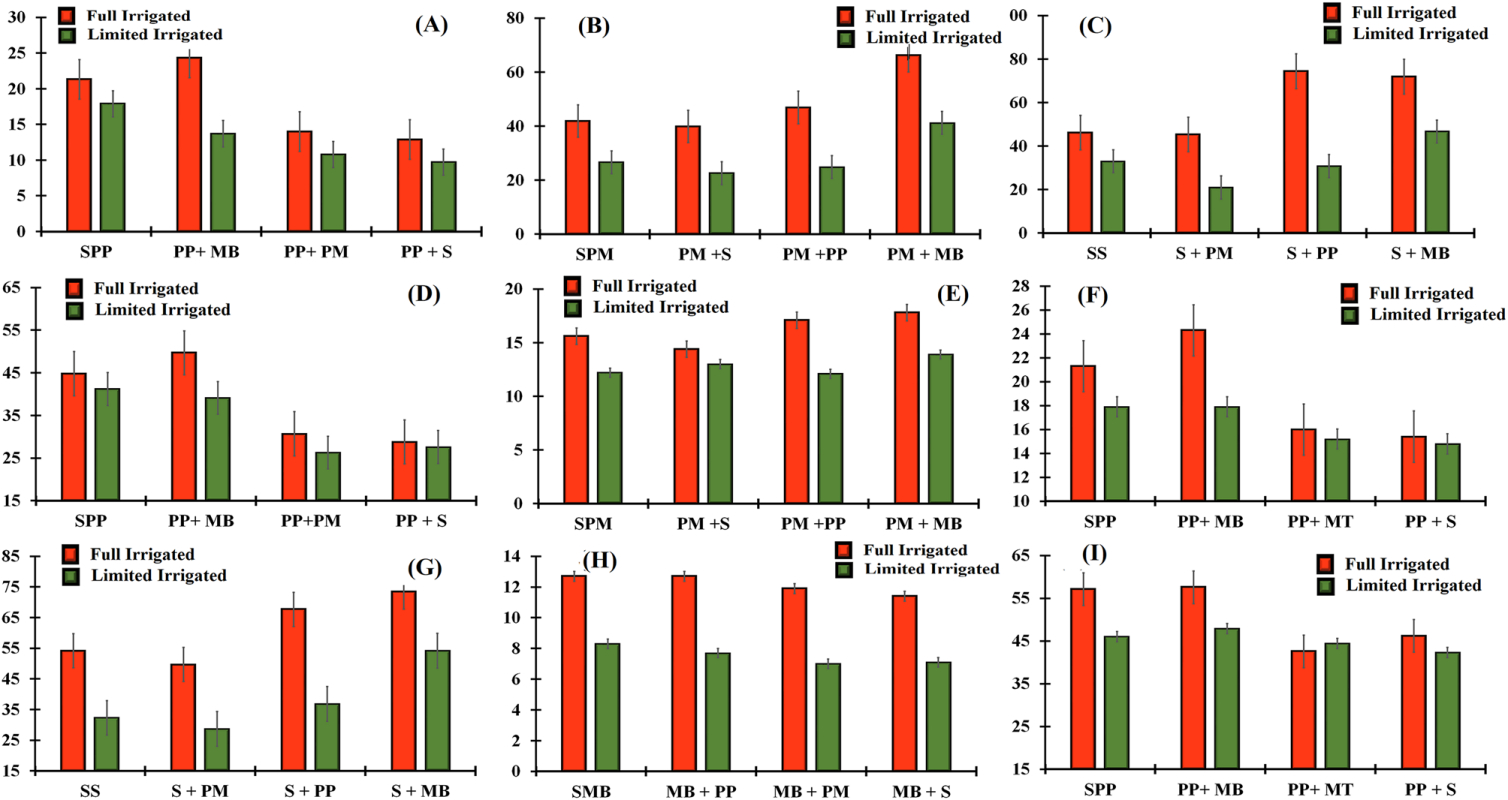
Leaves dry weight of pigeonpea (**A**), stem dry weight of pearl millet (**B**), stem dry weight of sorghum (**C**), stem dry weight of pigeonpea (**D**) at anthesis, respectively, leaves dry weight of pearl millet (**E**), leaves dry weight of pigeonpea (**F**), stem dry weight of sorghum (**G**), stem dry weight of mungbean (**H**), stem dry weight of pigeonpea (**I**) at physiological maturity.

Leaf dry weight (g plant^−1^) of millet, sorghum, mungbean and pigeonpea at anthesis were significantly affected by irrigation and intercropping (Table 5). All crops grown under full irrigated condition produced higher LDW plant^−1^ at anthesis than limited irrigated conditions. Both cereals, millet and sorghum intercropped with mungbean or with pigeon pea, produced maximum LDW plant^−1^ at anthesis than when intercropped with each other or grown as sole crops (Table 6A,B). Pigeonpea intercropped with mungbean or sown as sole crop produced higher LDW plant^−1^ under both water regimes, while the lowest LDW was recorded when pigeonpea was intercropped with sorghum under limited water condition (Fig. 4A). The planned mean comparison indicated that cereals intercropped with legumes produced higher LDW plant^−1^ at anthesis than intercropped with cereals. In contrast, legume intercropped with legume produced higher LDW plant^−1^ at anthesis than legumes intercropped with cereals. Moreover, both cereals produced higher LDW at anthesis in intercropping than sole cropping, while legumes produced maximum LDW plant^−1^ at anthesis in sole cropping than in intercropping. Millet and/or sorghum intercropped with mungbean produced higher LDW plant^−1^ at anthesis than intercropped with pigeonpea (Table 6C,D).

**Table 5:** Dry matter partitioning (g plant^−1^) at anthesis of summer crops as affected by intercropping and irrigation regimes.

Intercropping	Leaf	Stem	Head	Intercropping	Leaf	Stem	Head
Sole millet	19.7 b	34.3 b	11.5 bc	Sole sorghum	22.0 b	39.6 c	10.6 c
Millet + Sorghum	18.8 c	31.3 c	11.1 c	Sorghum + Millet	21.4 b	33.2 d	10.8 c
Millet + Pigeon pea	20.8 a	35.9 b	12.0 b	Sorghum + Pigeon pea	22.7 ab	52.6 b	11.8 b
Millet + Mungbean	21.6 a	53.6 a	13.0 a	Sorghum + Mungbean	23.5 a	59.3 a	12.6 a
Full irrigation	22.2	48.8	13.6	Full irrigation	24.4 a	59.5 a	13 a
Limited irrigation	18.3	28.8	10.2	Limited irrigation	20.4 b	32.9 b	9.9 b
Irrigation	**	*	*	Irrigation	*	*	*
LSD for intercropping	1	2.7	0.9	LSD for intercropping	1.4	4.3	0.7
LSD for interaction	ns	3.8	Ns	LSD for interaction	ns	6.1	ns
Intercropping	Leaf	Stem	Head	Intercropping	Leaf	Stem	Head
Sole mungbean	4.2 a	6.0 a	1.6 a	Sole pigeon pea	19.6 a	43.0 a	2.1 c
Mungbean + Pigeon pea	4.0 a	6.0 a	1.4 b	Pigeon Pea + Mungbean	19.0 a	44.4 a	3.0 a
Mungbean + Millet	3.9 a	5.6 ab	1.3 bc	Pigeon pea + millet	12.4 b	28.5 b	2.4 b
Mungbean + Sorghum	3.0 b	5.2 b	1.2 c	Pigeon Pea + sorghum	11.3 c	28.2 b	1.7 d
Full irrigation	5.3 a	8	1.5	Full irrigation	18.1	38.5	2.6
Limited irrigation	2.2 b	3.4	1.4	Limited irrigation	13	33.5	2
Irrigation	*	*	Ns	Irrigation	*	*	*
LSD for intercropping	4	0.4	0.2	LSD for intercropping	1	2	0.2
LSD for interaction	0.6	ns	Ns	LSD for interaction	ns	2.9	ns

**Table 6:** Pre-planned comparison of different intercropping systems at anthesis of summer crops as affected by intercropping and irrigation regimes.

**Leaves Dry weight (g) plant**^−1^	Pearl millet	Sole vs	Intercrop	Sig	ML + MB Vs	ML + PP	Sig	ML + SR	ML + PP/MB	Sig
	**(A)**	19.7	20.4	ns	21.6	20.8	ns	18.8	21.2	**
	Sorghum	Sole vs	Intercrop	Sig	S + MB Vs	S + PP	Sig	S + ML Vs	S + PP/MB	Sig
	**(B)**	22	22.5	ns	23.5	22.7	ns	21.4	23.1	**
	Mungbean	Sole vs	Intercrop	Sig	MB + ML Vs	MB + SR	Sig	MB + PP Vs	MB + ML/SR	Sig
	**(C)**	4.2	3.6	**	3.9	3	**	4	3.5	**
	Pigeonpea	Sole vs	Intercrop	Sig	PP + ML Vs	PP + SR	Sig	PP + MB Vs	PP + ML/SR	Sig
	**(D)**	19.6	14.2	**	12.4	11.3	*	19	11.8	**
**Stem Dry weight (g) plant**^−1^	Pearl millet	Sole vs	Intercrop	Sig	ML + MB Vs	ML + PP	Sig	ML + SR Vs	ML + PP/MB	Sig
	**(E)**	34.3	40.3	**	53.6	35.9	**	31.3	44.8	**
	Sorghum	Sole vs	Intercrop	Sig	S + MB Vs	S + PP	Sig	S + ML Vs	S + PP/MB	Sig
	**(F)**	39.6	48.4	**	59.3	52.6	**	33.2	55.9	**
	Mungbean	Sole vs	Intercrop	Sig	MB + ML Vs	MB + SR	Sig	MB + PP Vs	MB + ML/SR	Sig
	**(G)**	6	5.6	*	5.6	5.2	*	6	5.4	**
	Pigeonpea	Sole vs	Intercrop	Sig	PP + ML Vs	PP + SR	Sig	PP + MB Vs	PP + ML/SR	Sig
	**(H)**	43	33.7	**	28.2	28.5	ns	44.4	28.3	**
**Head/Pod Dry weight (g) plant**^−1^	Pearl millet	Sole vs	Intercrop	Sig	PM + MB Vs	PM + PP	Sig	PM + SR Vs	PM + PP/MB	Sig
	**(I)**	11.5	12	ns	13	12	*	11.1	12.5	**
	Sorghum	Sole vs	Intercrop	Sig	S + MB Vs	S + PP	Sig	S + PM Vs	S + PP/MB	Sig
	**(J)**	10.6	11.7	**	12.6	11.8	*	10.8	12.2	**
	Mungbean	Sole vs	Intercrop	Sig	MB + PM Vs	MB + SR	Sig	MB + PP Vs	MB + PM/SR	Sig
	**(K)**	1.6	1.3	**	1.3	1.2	ns	1.4	1.3	*
	Pigeonpea	Sole vs	Intercrop	Sig	PP + PM Vs	PP + SR	Sig	PP + MB Vs	PP + PM/SR	Sig
	**(L)**	2.1	2.4	**	2.4	1.7	**	3	2.1	**

Where ns, ** stands for non-significant and significant at 1% level of probability, respectively. PM, MB, S and PP stand for millet, mungbean, sorghum and pigeon pea, respectively. Means in the same category are not significantly different if followed by at least one common letter at (*P* ≤ 0.05) level.

#### Stem dry weight (g plant^−1^) at anthesis

Stem dry weight (g plant^−1^) at anthesis of millet, sorghum, mungbean and pigeonpea at anthesis were significantly affected by irrigation and intercropping and are presented in Table 5. Under full irrigated condition, all crops produced higher SDW plant^−1^ at anthesis than limited irrigated condition. Both millet and sorghum intercropped with mungbean produced maximum SDW plant^−1^ at anthesis, while lowest SDW plant^−1^ for both cereals was recorded when intercropped with each other (Fig. 4B,C), respectively. Pigeon pea intercropped with mungbean or sown as sole crop produced higher SDW plant^−1^ at anthesis (Fig. 4D) than when intercropped with millet and sorghum. The planned mean comparison indicated that cereals intercropped with legumes produced maximum SDW plant^−1^ at anthesis than intercropped with cereals (Table 6E,F). In contrast, legumes intercropped with legumes produced higher SDW plant^−1^ at anthesis than legumes intercropped with cereals (Table 6G,H). Moreover, millet and/or sorghum intercropped with mungbean produced higher SDW plant^−1^ at anthesis than intercropped with pigeon pea.

#### Head/pods dry weight (g plant^−1^) at anthesis

Head/pods dry weight (g plant^−1^) of millet, sorghum, mungbean and pigeon at anthesis are shown in Table 5 and this parameter was significantly affected by irrigation and intercropping. All crops grown under full irrigated condition produced higher H/PDW plant^−1^ at anthesis than limited irrigated condition. Both cereals, millet and sorghum intercropped with mungbean or with pigeon pea had produced maximum H/PDW plant^−1^ at anthesis than intercropped with each other (Table 6I,J), respectively. Mungbean intercropped with pigeon pea or pigeon pea intercropped with mungbean or sown as sole crop produced higher H/PDW plant^−1^ than intercropped with cereals (Table 6K,L). The planned mean comparison indicated that cereals intercropped with legumes produced higher H/PDW plant^−1^ at anthesis than when intercropped with other cereals. In contrast, legumes intercropped with legumes produced higher H/PDW plant^−1^ at anthesis than legumes intercropped with cereals crops (Table 6K,L). Moreover, all crops produced comparatively higher H/PDW at anthesis in intercropping than mono-cropping, except for sole mungbean. Millet and/or sorghum intercropped with mungbean produced high HDW at anthesis than intercropped with pigeon pea.

#### Leaf dry weight (g plant^−1^) at physiological maturity

Data about leaves dry weight (LDW) plant^−1^ at PM of millet, sorghum, mungbean and pigeon are shown in Table 7. Both irrigation and intercropping were highly significantly affected LDW plant^−1^ at PM. All crops grown under full irrigated condition produced higher LDW plant^−1^ at PM than under limited irrigated condition. Both cereals, millet and sorghum intercropped with mungbean or with pigeon pea produced maximum LDW plant^−1^ than intercropped with each other (Table 8A,B), respectively. Mungbean intercropped with pigeon pea or pigeon pea intercropped with mungbean or sown as sole crops produced higher LDW plant^−1^ than intercropped with millet and sorghum (Table 8C,D), respectively. The planned mean comparison revealed that cereals intercropped with legumes produced higher LDW than when intercropped with cereals. In contrast, legume intercropped with legume produced higher LDW than legume intercropped with cereal crops. Furthermore, both cereals produced higher LDW in intercropping than mono-cropping. In contrast, legumes produced maximum LDW in sole cropping than intercropping. Millet intercropped with mungbean produced higher LDW than when intercropped with pigeon pea under both water regimes (Fig. 4E). Similarly, pigeonpea intercropped with pearl millet produced comparatively higher LDW than intercropped with sorghum (Fig. 4F).

**Table 7:** Dry matter partitioning (g plant^−1^) at physiological maturity of summer crops as affected by intercropping and irrigation regimes.

Intercropping	Leaf	Stem	Head	Intercropping	Leaf	Stem	Head
Sole millet	13.9 bc	38.3 b	23.7 ab	Sole sorghum	14.3 c	43.2 c	26.7 b
Millet + Sorghum	13.7 c	37.9 b	22.3 b	Sorghum + Millet	14.9 bc	39.2 d	26.0 b
Millet + Pigeon pea	14.6 b	40.1 b	24.9 a	Sorghum + Pigeon pea	15.6 b	52.2 b	28.9 a
Millet + Mungbean	15.8 a	56.5 a	24.1 a	Sorghum + Mungbean	16.5 a	63.8 a	29.4 a
full irrigation	16.2 a	52.6 a	27.7 a	full irrigation	17.6 a	61.2 a	30.5 a
Limited irrigation	12.8 b	33.8 b	19.8 b	Limited irrigation	13.1 b	38.0 b	25.0 b
Irrigation	*	*	*	Irrigation	*	*	*
LSD for intercropping	0.8	3.4	1.7	LSD for intercropping	0.8	2.7	1.5
LSD for interaction	ns	5.6	ns	LSD for interaction	ns	ns	ns
Sole mungbean	6.6 a	10.5 a	7.7 a	Sole pigeon pea	19.6 b	51.5 a	15.2 b
Mungbean + Pigeon pea	6.3 a	10.2 a	7.5 a	Pigeon pea + Mungbean	21.1 a	52.8 a	19.6 a
Mungbean + Millet	6.2 a	9.4 b	7.4 a	Pigeon pea + Millet	15.6 c	43.5 b	14.0 b
Mungbean + Sorghum	5.3 b	9.2 b	7.0 b	Pigeon pea + Sorghum	15.1 c	44.3 b	11.2 c
full irrigation	7.6 a	12.2 a	8.4 a	full irrigation	19.2 a	50.9 a	17.8 a
Limited irrigation	4.6 b	7.5 b	6.4 b	Limited irrigation	16.5 b	45.2 b	12.2 b
Irrigation	*	*	*	Irrigation	*	*	*
LSD for intercropping	0.6	0.7	0.4	LSD for intercropping	1.4	2.7	1.9
LSD for interaction	ns	1.2	ns	LSD for interaction	ns	ns	ns

**Table 8:** Pre-planned comparison of different intercropping systems at physiological maturity of winter crops as affected by intercropping and irrigation regimes.

Leaves Dry weight (g) plant^−1^	Pearl millet	Sole vs	Intercrop	Sig	ML + MB	ML + PP	Sig	ML + SR Vs	ML + PP/MB	Sig
	**(A)**	13.9	14.7	*	15.8	14.6	**	13.7	15.2	**
	Sorghum	Sole vs	Intercrop	Sig	S + MB Vs	S + PP	Sig	S + ML Vs	S + PP/MB	Sig
	**(B)**	14.3	15.7	**	16.5	15.6	*	14.9	16	**
	Mungbean	Sole vs	Intercrop	Sig	MB + ML Vs	MB + SR	Sig	MB + PP Vs	MB + ML/SR	Sig
	**(C)**	6.6	5.9	**	6.2	5.3	**	6.3	5.8	*
	Pigeonpea	Sole vs	Intercrop	Sig	PP + ML Vs	PP + SR	Sig	PP + MB Vs	PP + ML/SR	Sig
	**(D)**	19.6	17.3	**	15.6	15.1	ns	21.1	15.3	**
Stem Dry weight (g) plant^−1^	Pearl millet	Sole vs	Intercrop	Sig	ML + MB Vs	ML + PP	Sig	ML + SR Vs	ML + PP/MB	Sig
	**(E)**	38.3	44.8	**	56.5	40.1	**	37.9	48.3	**
	Sorghum	Sole vs	Intercrop	Sig	S + MB Vs	S + PP	Sig	S + ML Vs	S + PP/MB	Sig
	**(F)**	43.2	51.7	**	63.8	52.2	**	39.2	58	**
	Mungbean	Sole vs	Intercrop	Sig	MB + ML Vs	MB + SR	Sig	MB + PP Vs	MB + ML/SR	Sig
	**(G)**	10.5	9.6	**	9.4	9.2	ns	10.2	9.3	**
	Pigeonpea	Sole vs	Intercrop	Sig	PP + ML Vs	PP + SR	Sig	PP + MB Vs	PP + ML/SR	Sig
	**(H)**	51.5	46.9	**	43.5	44.3	ns	52.8	43.9	**
Head/Pod Dry weight (g) plant^−1^	Pearl millet	Sole vs	Intercrop	Sig	ML + MB Vs	ML + PP	Sig	ML + SR Vs	ML + PP/MB	Sig
	**(I)**	23.7	23.8	Ns	24.1	24.9	ns	22.3	24.5	**
	Sorghum	Sole vs	Intercrop	Sig	S + MB Vs	S + PP	Sig	S + ML Vs	S + PP/MB	Sig
	**(J)**	26.7	28.1	*	29.4	28.9	ns	26	29.2	**
	Mungbean	Sole vs vs	Intercrop	Sig	MB + ML Vs	MB + SR	Sig	MB + PP Vs	MB + ML/SR	Sig
	**(K)**	7.7	7.3	*	7.4	7	ns	7.5	7.2	*
	Pigeonpea	Sole vs	Intercrop	Sig	PP + ML Vs	PP + SR	Sig	PP + MB Vs	PP + ML/SR	Sig
	**(L)**	15.2	15	Ns	14	11.2	**	19.6	12.6	**

Where *, ** stands for significant at 5 and 1% level of probability, respectively. ML, MB, SR and PP stand for millet, mungbean, sorghum and pigeon pea and W, B, FB, R, stand for wheat, barley, fababean and rapeseed, respectively. Means in the same category are not significantly different if followed by at least one common letter at (*P* ≤ 0.05) level.

#### Stem dry weight (g plant^−1^) at physiological maturity

Data regarding stem dry weight (SDW) of millet, sorghum, mungbean and pigeon pea at PM are presented in Table 7 and this parameter was significantly affected by irrigation and intercropping. Under full irrigated condition, all crops produced higher SDW plant^−1^ than limited irrigated condition. Both cereals intercropped with mungbean produced maximum SDW plant^−1^, followed by intercropped with pigeon pea, while lowest SDW plant^−1^ of both cereals were observed when intercropped with each other (Fig. 4G). Mungbean intercropped with pigeon pea (Fig. 4H) or pigeon pea intercropped with mungbean or sown as sole crops (Fig. 4I) produced higher SDW plant^−1^ than when intercropped with millet/sorghum. The planned mean comparison specified that cereals intercropped with legumes produced maximum SDW plant^−1^ than intercropped with cereals (Table 8E,F). In contrast, legumes intercropped with cereals produced less SDW plant^−1^ than legumes intercropped with legumes (Table 8G,H). Moreover, millet and/or sorghum intercropped with mungbean produced higher SDW plant^−1^ than intercropped with pigeon pea.

#### Head/pods dry weight (g plant^−1^) at physiological maturity

Data on head/pods dry weight (H/PDW) plant^−1^ of millet, sorghum, mungbean and pigeon at PM are presented in Table 7. H/PDW all of crops at PM was significantly affected by irrigation and intercropping. All crops grown under full irrigated condition produced higher H/PDW plant^−1^ than under limited irrigated condition. Both cereals, millet and sorghum intercropped with mungbean or pigeon pea produced maximum H/PDW plant^−1^ than other intercrops (Table 8I,J). Mungbean intercropped with pigeon pea or pigeon pea intercropped with mungbean or sown as sole crops produced higher H/PDW plant^−1^ than intercropped with cereals (Table 8K,L). The planned mean comparison indicated that cereals intercropped with legumes produced higher H/PDW plant^−1^ than intercropped with cereals. In contrast, legumes intercropped with legumes produced higher H/PDW plant^−1^ than legume intercropped with cereal crops.

## Discussion

Sorghum and pearl millet intercropped with mungbean and pigeon produced higher CGR and this might be due to more nitrogen availability from mungbean and pigeonpea and less intra competition^28^ because of the low stature of mungbean and pigeonpea^32,33^. However, pigeonpea showed a strong competition with both of the cereals probably due to their strong deep root system^34–36^ and highly branched stature, which can adopt to various conditions by modification in plant canopy. In contrast, the CGR of mungbean and pigeonpea was suppressed by the both cereals^37,38^ (sorghum and pearl millet). Both cereals have strong root system and have high leaf area and taller in nature, which shade the low stature mungbean, and as a result spurred the growth of mungbean^39^. In the case of sorghum grown as sole or intercropped with pearl millet decreasing their growth as compared with intercropped with legumes might be due to very high inter and intra species competition^28^. In the case of winter crops, the CGR of wheat /barley was enhanced by the intercropping of fababean probably due to nitrogen transfer by fababean^40–42^ as compared with wheat intercropped with barley^43^ or rapeseed. Similarly, a decrease in barley CGR in all intercrops might be due to allelopathic effect of barley in other intercrops^28^. Dry matter yield at different growth stages as well as DM partitioning into leaf, stem and spike/pods/siliques were statistically found significant for irrigation, intercropping and their interface. All parameters regarding DM at different growth stages and DM partitioning into different plant parts showed that full irrigation gave higher DM yield compared to limited irrigation. Among intercropping, the highest DM of leaves, stems, spikes at anthesis and maturity was maximum when wheat + fababean were intercropped. Interaction of irrigation and intercropping revealed that all combinations of crops under fully irrigated conditions produced the highest DM partitioning with maximum in wheat + fababean. Likewise results for DM partitioning was concluded by^44–46^ that intercropping system significantly affected DM of wheat^47,48^ stated that DM yield was affected by different intercropping systems and this is in line with our discoveries. Plausible explanation for more DM yield of cropping system may be due to the ability of component crops to exploit many layers of soil due to different roots depth in search for resources and presenting no competition. This results in good resource utilization such as light, nutrients and water^19,44,49^.

The DM partitioning into stem, leaves and spike at anthesis and maturity were statistically significant for irrigation and intercropping. Leaf dry weight (gm^−2^), stem dry weight and spike dry weight have more dry weight under no stress associated to stress condition^50–52^. For different patterns of cropping, significant results for these parameters were obtained when barley was planted with legume with the only exception of spike dry weight at anthesis that was maximum for pure barley stand. These results are in line with those obtained by^53–55^ who reported that intercropping greatly affected fresh yield at stages of wheat when intercropped with brassica. They also reported that interaction of cropping system and seed ratio was found significant for fresh fodder yield. Total DM yield at different growth stages as well as DM partitioning into leaf, stem and spike/pods/siliques were statistically found significant for irrigation, intercropping and their interface.

The DM partitioning and accumulation were high under high moisture condition, which lead to higher yield. The present results are similar with the findings of^50,56–60^ who reported that grain weight was reduced under moisture stress condition. Stem, leaf and head/pod dry weights plant^−1^ of millet, sorghum, mungbean and pigeon pea at anthesis and at physiological maturity were significantly affected by irrigation and intercropping. Maximum stem, leaf, and head/pods weight were higher under full irrigated conditions than limited irrigated conditions.^61^ reported that increases in moisture contents increase stem weight. These results are also in line with those of^62^. They reported that leaf dry weight was high at high moisture condition. Higher pods dry weight in mungbean might be due to more translocation of assimilate towards pod and grains^63^. Water deficit irrigation decreased leaf area and LAI as a result of decreased photosynthesis and transpiration rate, which lead to less DM production, and hinder the translocation of assimilate towards the sink^28,64^. The present results are in line with the work of^50,64–66^. These authors reported less DM accumulation in water stress condition^41^ reported that in water stress conditions, plant start accumulation of assimilate in root and stem while in well water condition assimilate diverted into the reproductive parts. Cereals produced maximum leaf, stem and head weight in intercropping with legumes^67^. The increase of cereal leaf stem and head weights was probably due to the more space availability, more sunshine, large leaf area, more soil resources and less competition because of the small stature of legumes as compared with sorghum and millet. At physiological maturity dry weight of stem and head was high than at anthesis because of more DM accumulation, in stem and head, but leaf weight was decreased slightly, because of more translocation of assimilate toward pods during grain filling. In contrast, legumes produced higher DM when intercropped with legume as compared to intercropped with sorghum and millet. This might be due to the suppressive effect of sorghum and millet on mungbean and pigeon pea, as a result, DM production and partition drastically decreased in legume crops when intercropped with cereal^28^. The results are also in line with^28,68^, who reported that DM plant^−1^ of pigeon pea was high when intercropped with urdbean than intercropped with sorghum.

## Conclusion

From the results it was concluded that cereal/legume intercropping particularly wheat + fababean in winter, and sorghum + pigeon or sorghum + mungbean in summer are the most productive intercropping systems under full and limited irrigation regimes.
